# Coronary artery segmentation under class imbalance using a U-Net based architecture on computed tomography angiography images

**DOI:** 10.1038/s41598-021-93889-z

**Published:** 2021-07-14

**Authors:** Li-Syuan Pan, Chia-Wei Li, Shun-Feng Su, Shee-Yen Tay, Quoc-Viet Tran, Wing P. Chan

**Affiliations:** 1 Department of Electrical Engineering, National Taiwan University of Science and Technology, Taipei, 10607 Taiwan; 2 Department of Radiology, Wan Fang Hospital, Taipei Medical University, Taipei, 10607 Taiwan; 3 Department of RadiologySchool of MedicineCollege of Medicine, Taipei Medical University, Taipei, 10607 Taiwan; 4 Medical Innovation Development Center, Wan Fang Hospital, Taipei Medical University, Taipei, 10607 Taiwan

**Keywords:** Anatomy, Cardiology

## Abstract

Coronary artery disease is caused primarily by vessel narrowing. Extraction of the coronary artery area from images is the preferred procedure for diagnosing coronary diseases. In this study, a U-Net-based network architecture, 3D Dense-U-Net, was adopted to perform fully automatic segmentation of the coronary artery. The network was applied to 474 coronary computed tomography (CT) angiography scans performed at Wanfang Hospital, Taiwan. Of these, 10% were used for testing. The CT scans were divided into patches of 16 original high-resolution slices. The slices were overlapped between patches to take advantage of surrounding imaging information. However, an imbalance between the foreground and background presents a challenge in smaller-object segmentation such as with coronary arteries. The network was optimized and achieved a promising result when the focal loss concept was adopted. To evaluate the accuracy of the automatic segmentation approach, the dice similarity coefficient (DSC) was calculated, and an existing clinical tool was used. The subjective ratings of three experienced radiologists were used to compare the two ratings. The results show that the proposed approach can achieve a DSC of 0.9691, which is significantly higher than other studies using a deep learning approach. In the main trunk, the results of automatic segmentation agree with those of the clinical tool; they were significantly better in some small branches. In our study, automatic segmentation tool shows high-performance detection in coronary lumen vessels, thereby providing potential power in assisting clinical diagnosis.

## Introduction

Coronary CT angiography (CCTA) provides detailed imaging and can deliver precise analyses and prognostic information for diagnoses (Leipsic et al.^1^). Extracting coronary arteries from CCTA scans is a critical step for analyzing those images. A typical first step is extracting the coronary artery centerline allowing reconstruction of the coronary artery area based on its position and the predicted radius. Several automatic approaches have been proposed for extracting the coronary artery centerline^2–5^. In contrast to centerline extraction, segmentation of the coronary arteries improves visualization and quantification of the vessels. Manual segmentation of CCTA scans by radiologists is time-consuming because only 2D slices of the CT images are available after image acquisition. Extracting the coronary arteries from 2D images relies on expert knowledge, but even the experts make errors because of the tedious nature of the work. Diagnosing coronary artery diseases depends primarily on computed tomography (CT) images, and therefore a high-quality fully automatic segmentation approach for defining the coronary arteries is essential.

Deep learning, especially the convolutional neural network (CNN), has recently shown capabilities in an extensive range of medical image analyses (Litjens et al.^6^, 2014, 2017). A deep CNN network architecture, U-Net^7^, has shown promising results in automatic segmentation for a variety of medical applications^8–14^. In recent years, researchers have studied coronary artery reconstruction based on deep learning approaches. For example, Wolterink et al.^5^ proposed using a 3D CNN classifier to predict the direction and radius of an artery at any given point in a CCTA image. Kjerland et al.^15^ proposed generating volume data as a training set from centerline data and considered a two-stream CNN architecture that could use different scales of input to perform a fully automatic segmentation task. Huang et al.^16^ employed a basic 3D U-Net convolutional network for coronary artery segmentation using a small amount of real-world data. Chen et al.^17^ incorporated a vessel map into CT angiography images using the proposed 3D multi-channel U-Net. However, these studies have relatively limited performance and are evaluated using a small dataset for testing [e.g., the dice similarity coefficient (DSC) has been shown to be 0.5975^15^ in 1 case, 0.7146^16^ in 4 cases, and 0.8060^17^ in 4 cases (2 for validation, 2 for testing)]. The differences could be due to class imbalance, which was not considered.

The focus of this study was to perform high-quality fully automatic artery segmentation in a deep learning approach that can be used clinically. Here, a U-Net-based architecture, 3D Dense-U-Net^18^, was adopted with the focal loss^19^ function instead of the typical DSC loss to address class imbalance. Furthermore, rather than cropping images to accommodate hardware limitations faced in other deep learning approaches, all data were processed to maintain the original high resolution^18^ thus ensuring that the global information was sufficient. To validate the performance of this approach, the test data were evaluated not only using intersection over union (IoU) and DSC, but the results were compared with the clinical tool; subjective ratings of their performances were provided by three experienced radiologists.

## Materials and methods

### Data description

This study was performed in accordance with relevant guidelines and was conducted after approval by the Taipei Medical University-Joint Institutional Review Board (N201710005). Informed consent was waived because of its retrospective nature.

CCTA scans were acquired using a dual-source CT scanner at the Department of Radiology, Wanfang Hospital, Taipei Medical University, Taiwan (Somatom Definition; Siemens Medical Solutions, Forchheim, Germany). Those obtained from 2013 to 2019 were retrospectively compared against our inclusion criteria: (1) full information about the entire coronary tree was collected and (2) the patient was aged more than 20 years but less than 85 years yielding 474 qualified scans out of 480 scans (six unqualified scans were excluded). All scans were collected using a tube voltage of 120 kVp and a maximum tube current of 900 mA. Images were reconstructed to a mean voxel size of 0.32 × 0.32 × 0.7 mm^3^, and the margins of two major coronary arteries [the left coronary artery (LCA) and the right coronary artery (RCA)] and all their branches were manually annotated by consensus between three radiology technologists and approved by another radiologist experienced in cardiovascular imaging. These approved annotated images were used as the ground truth. In this study, only non-stenotic CCTA dataset were included for developing segmentation algorithm. The scans were divided into a training set (432 scans) and a test set (42 scans). From the latter, 42 were used to compare against the existing semi-automatic clinical method (Syngo.via, Siemens).

### Preprocessing

**Figure 1:**
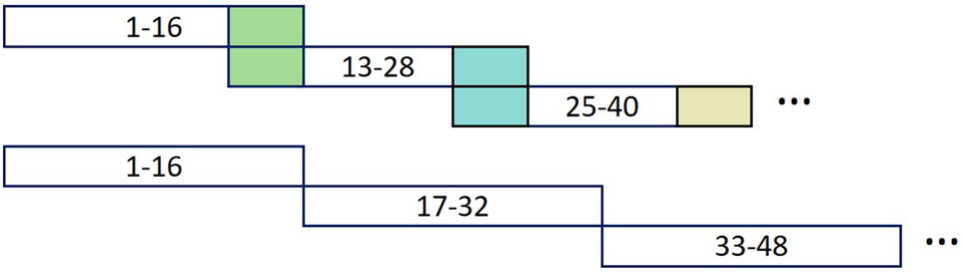
Examples of data preprocessing show the index numbers of the slices included in each patch (represented by a long rectangle). The training data (upper) were overlapped such that four slices were shared between one patch and the next. The test data (lower) were processed without overlapping.

Each scan was a 3D medical image of the coronary arteries at a resolution of 512 × 512 spanning multiple slices (usually between 140 and 220 slices). The memory in the graphics processing unit (GPU) could not accommodate all the slices at this resolution; therefore, each scan was divided into patches of 16 slices each maintaining the original resolution. This preprocessing approach assured that sufficient global information was retained in each slice and is a clear advantage versus transforming the data to a lower resolution. Slices between patches were overlapped, and correlated information between them was utilized so that every voxel of every image could be analyzed and compared with surrounding slices during training. Figure 1 shows four overlapped slices in the training data, but there was no need in test data.

### Network architecture

**Figure 2:**
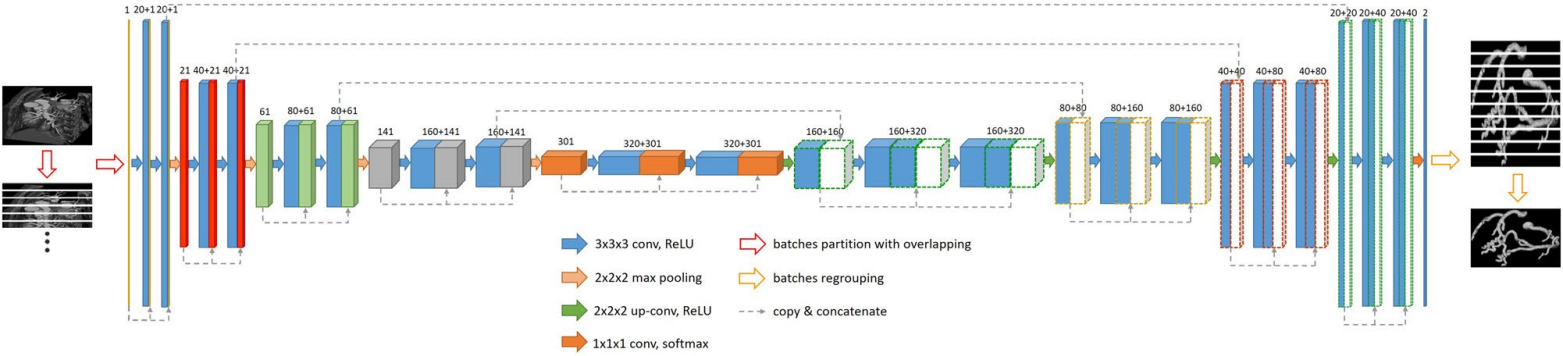
Flowchart of 3D Dense-U-Net. The input data is divided into patches that are then processed by the network. Each block represents a multi-channel feature map (channel number shown above the block). The network consists of down-sampling and up-sampling layers with concatenating parts. Each color within the dotted lines represents the same feature map concatenation. The network output is regrouped into the original 3D medical image.

U-Net is widely used for medical image segmentation by employing its convolutional encoder-decoder architecture. We used the improved version (3D Dense-U-Net^18^) to perform the residual^20^ and dense^21^ interconnections. This provides better results. A detailed network architecture is shown in Fig. 2. We believe that the Dense-U-Net architecture can provide high-quality results in coronary artery segmentation tasks.

### Weighted focal loss

Dense-U-Net was originally designed for segmentation of brain and spine data. The objects in the coronary artery area are much smaller and require a greater focus on the learning process. Focal loss concept^19^ is proposed for this kind of sparse data. Our research shows that this indeed achieves better performance.

In CCTA images, a huge imbalance between the coronary artery and the background area is seen, and most labels contain useless information. In most cases, the ratio between the area of the coronary artery and the area of the background is up to 1:400. This can confound the results when 3D Dense-U-Net is employed directly. The focal loss concept was first designed for object detection tasks where an extreme imbalance between the foreground and background classes is present. The class imbalance can cause inefficient network training because most locations are negative samples (background) and usually cannot contribute useful learning information. Additionally, negative samples can overwhelm the positive ones (arteries) during training because most loss values originate from negatives. This can be improved by employing the focal loss concept:

1 $$ FL(p,\;~y) = ~ - \alpha (1~ - ~p)^{\gamma} ~\;ylog(p) - ~(1~ - ~\alpha )p^{\gamma} ~(1~ - ~y)log(1~ - ~p) $$

where y is the ground truth (1 for arteries and 0 for background), p ∈ [0, 1] is the predicted probability that the network belongs to the positive class, α is a class weight for the positive class, and γ is a focusing parameter for adjusting the weight of well-classified samples. In this study, the class imbalance is resolved when α = 0.6 and γ = 2; misclassified samples are nearly eliminated.

## Results

**Figure 3:**
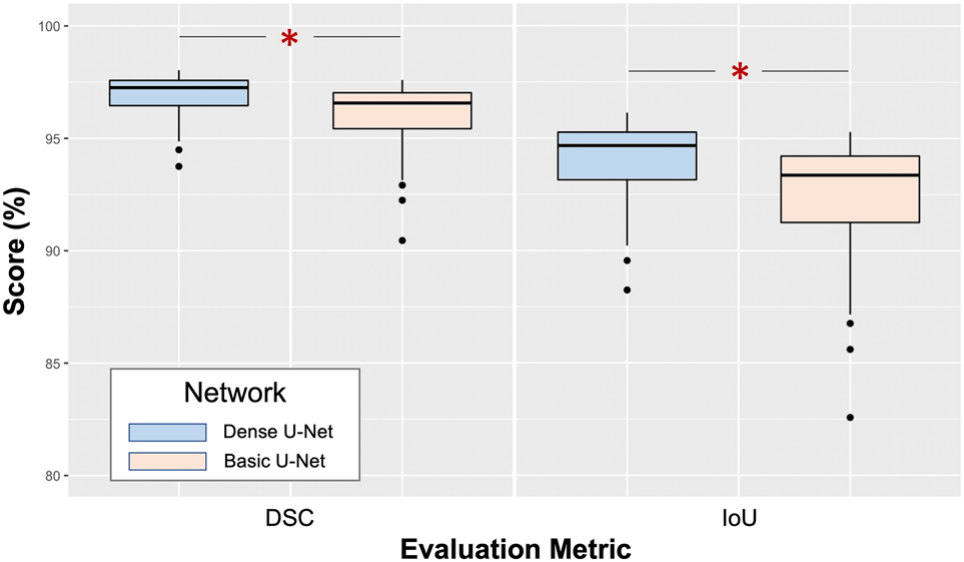
Comparison of the performances of basic U-Net and Dense-U-Net using the test data set. (*p < 0.01).

In this study of 474 coronary CT images collected at our institution, the test set of 42 scans was used to evaluate our results employing IoU and DSC; both are widely used in qualifying image detection and medical volume segmentation tasks. These metrics are defined as:

2 $$ IoU({\text{Y}},\;\hat{{\text{Y}}}) = \frac{{\left| {Y \cap \hat{Y}} \right|}}{{\left| {Y \cup \hat{Y}} \right|}} $$

3 $$ DSC({\text{Y}},~\;\hat{{\text{Y}}}) = \frac{{2\left| {Y \cap \hat{Y}} \right|}}{{\left| {Y\left|  +  \right|\hat{Y}} \right|}} $$

where Y denotes the expert’s annotation, and $\hat{{\text{Y}}}$ denotes the model’s prediction. In this study, Y and $\hat{{\text{Y}}}$ ∈ {0, 1}. The predicted results of the model were evaluated using 3D IoU and 3D DSC on our test set. The results using basic 3D U-Net were compared to those using 3D Dense-U-Net. Figure 3 shows the accuracy based on the test set demonstrating that IoU exceeds 94% and DSC is around 97% in more than half of the scans when 3D Dense-U-Net is employed; this performance is significantly better than that of the basic version (by paired t-test; p = 4.11 × 10^–13^). The averages for IoU and DSC using 3D Dense-U-Net were 94.03% and 96.91% compared to 92.40% and 96.03% when using basic U-Net (significance; p = 2.62 × 10^–12^).

**Figure 4:**
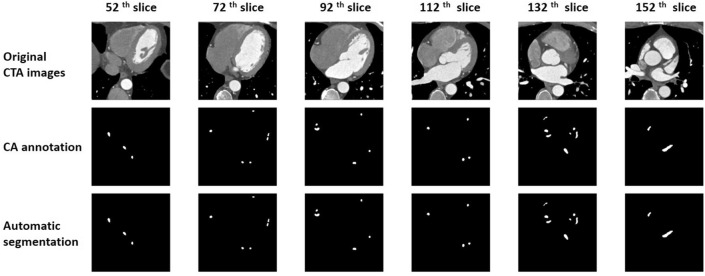
Annotation and automatic segmentation results are visualized in 2D using every 20th slice of the original images. The regions of the coronary artery are marked in white in both the CA annotation and automatic segmentation.

**Figure 5:**
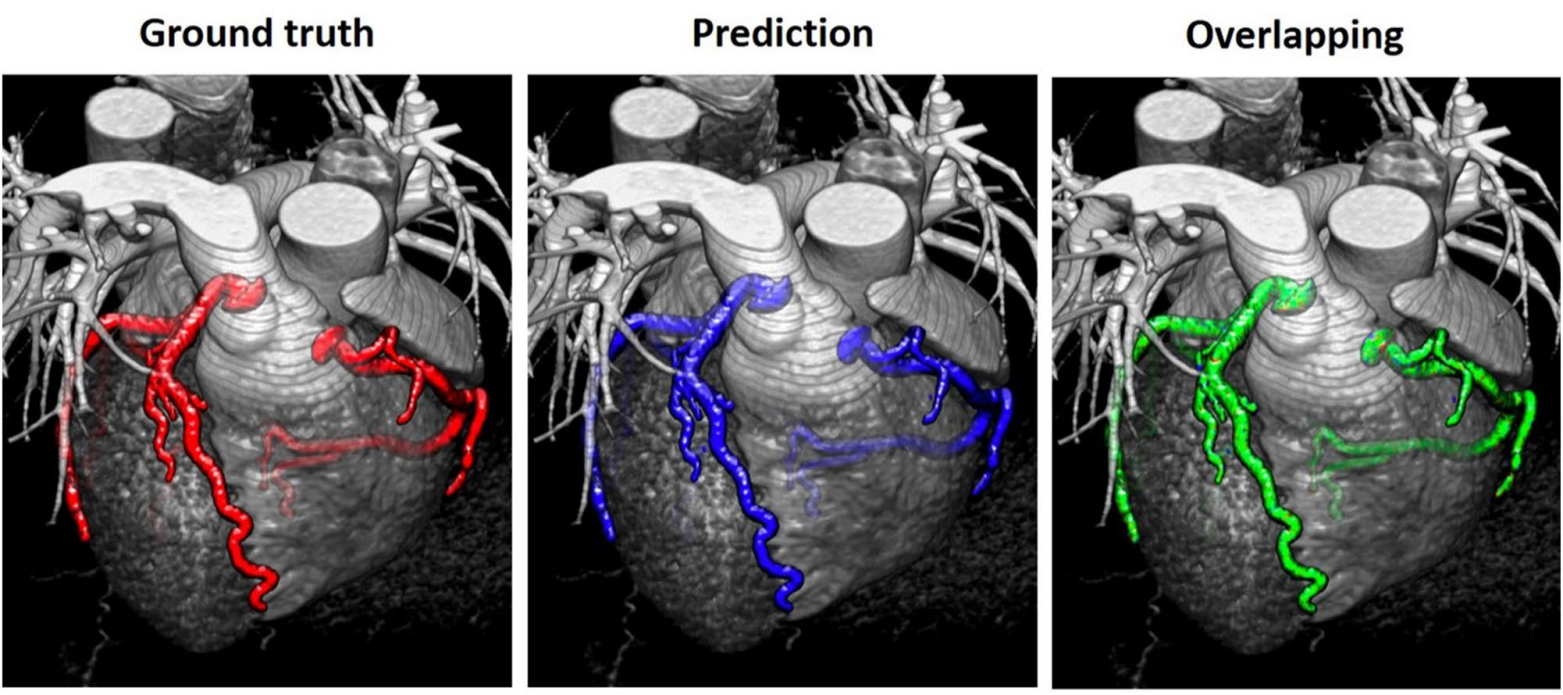
The 3D visualization of the CCTA image shows (from left to right) the ground truth (red), the predicted region of the arteries (blue), and an overlap of the two (green). In the overlap, most of the arteries are shown in green, but an extremely tiny bit of noise can be seen in blue and red.

The trained network was tested using the test set, and the experimental results were visualized in both 2D and 3D (Figs. 4, 5). Figure 4 shows part of the slices from the original CCTA images—both the ground truth and the network-predicted results. The images were sampled every 20 slices. Most of the predicted results were quite similar to the ground truth even when the regions of the coronary artery were small. Figure 5 shows the 3D visualizations. To clearly compare the results, the ground truth and the network prediction, each in its own color, were overlapped into a single image showing overlapping areas in a different color and areas not in agreement in their original colors. Most areas of the arteries are the same between the two. This shows that using the 3D Dense-U-Net architecture in combination with the focal loss concept can achieve a promising result when automatically segmenting CCTA images.

**Figure 6:**
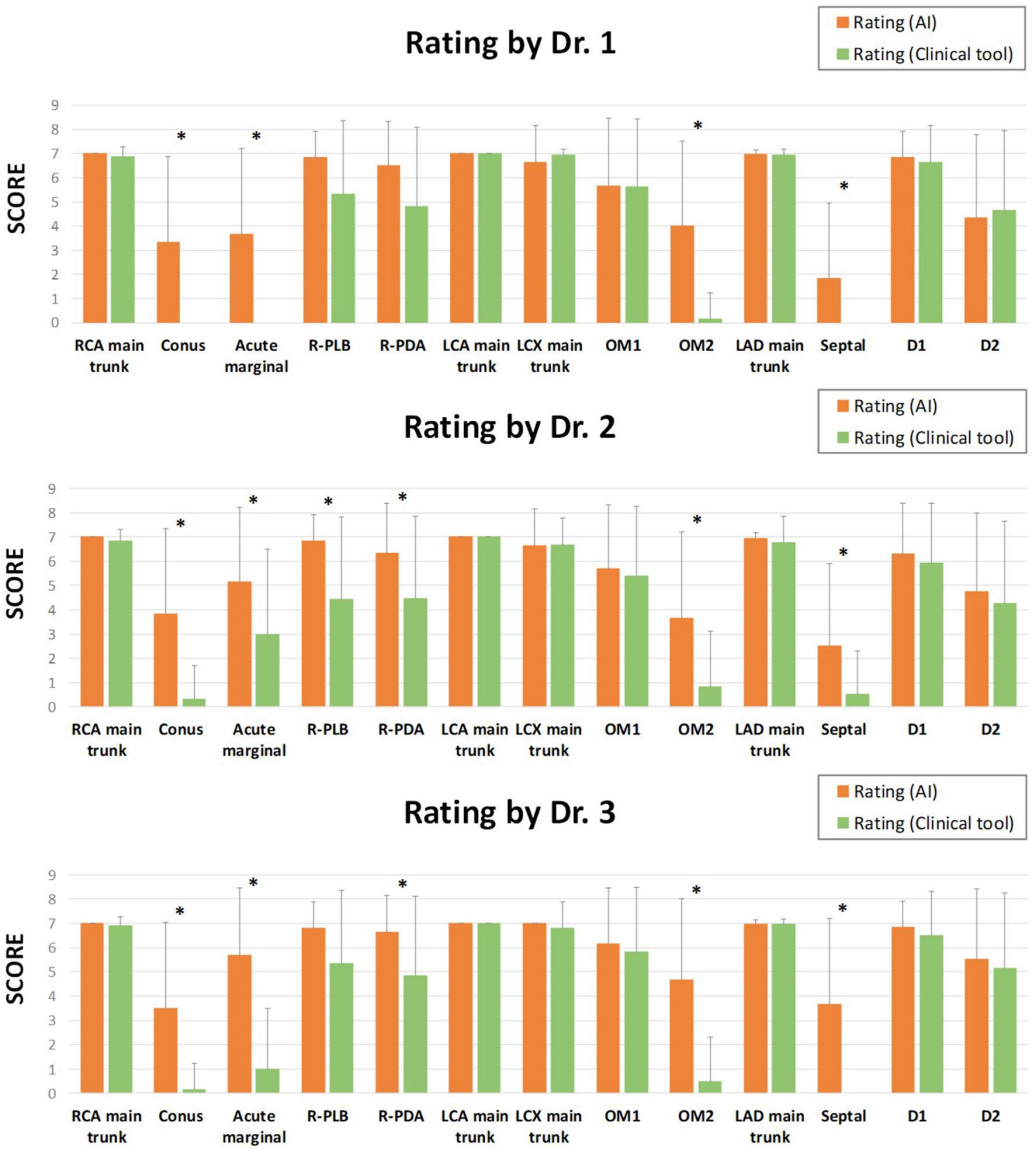
The subjective ratings of three experienced radiologists on the extraction of the coronary arteries by our automatic method and an existing clinical tool. The two were compared using a rating system on 13 branches: the main trunk of the right coronary artery (RCA), right conus artery, right acute marginal artery, right posterior interventricular branch (R-PLB), right posterior descending artery (R-PDA), main trunk of left coronary artery (LCA), main trunk of left circumflex coronary (LCX) arteries, obtuse marginal one artery (OM1), obtuse marginal two artery (OM2), main trunk of left anterior descending (LAD) arteries, left interventricular septal artery, the 1st diagonal branch (D1), and the 2nd diagonal branch (D2). Compared with the performance of the clinical tool, our method yielded relatively better scores in all extracted branches (*Bonferroni-corrected p < 0.05).

These results were compared to those from the existing semi-automatic clinical tool (Syngo.via, Siemens) based on coronary arterial centerline detection and which was applied to 42 clinical scans from the test set. The time taken for segmentation by our method (about 10–15 s) is almost equal to the time taken for segmentation by Siemens Syngo.via (about 12 s). The accuracy of arterial segmentation using the two approaches were subjectively scored by three experienced radiologists. They were asked to rate the outcomes of the segmentation images produced by our approach and via the use of the coronary arterial centerlines by a clinical tool on an 8-point scale (7, entirely correct; 6, 1 error; 5, 2 errors; 4, 3 errors; 3, 4 errors; 2, 5 errors; 1, 6 errors; and 0, more than 7 errors). Statistical analyses were performed using the R software program with paired t-test functionality version 3.5.1. A difference was considered statistically significant when the Bonferroni-corrected p-value was less than 0.05. Figure 6 shows the subjective ratings of the segmentation results by our approach and by the clinical tool. The results show that our approach is correct by the clinical tool in the main trunks and the main branches of the coronary arteries. Furthermore, the detection accuracy of our approach is significantly better for some small branches of the coronary arteries (Bonferroni-corrected p = 0.05).

## Discussion

We demonstrated that combining the U-Net based network with the focal loss concept indeed can achieve an excellent result when applied to scans from our CCTA database. Furthermore, the network was validated on a relatively larger test set to ensure that it can be generalized to unseen real-world data. To confirm that our research can be used clinically, the network prediction was compared with an existing clinical tool (Syngo.via, Siemens). The segmentation scores show that this new approach is significantly better than using the existing clinical tool in the automatic separation of the coronary lumen arteries.

To date, coronary artery segmentation studies using deep learning approaches have given relatively limited performances^15–17^. One reason could be that the class imbalance was not considered in most of these studies. Chen et al.^17^ attempted to balance the number of samples between the vessel areas and the background regions by data argumentation. The result was relatively better than those of the two approaches not considering the imbalance^15,16^. It is clear that the network cannot provide good performance without considering the class imbalance. Buda et al.^22^ demonstrated that the impact of class imbalance on classification tasks of CNNs is detrimental. In practice, the small volumes of the arteries are the primary challenge because the network cannot learn when an abundance of useless information is in the learning dataset. Using the loss function concept is more critical for improving segmentation of the coronary artery region compared to other methods. It has been shown that oversampling from minor classes of data can lead to overfitting^23,24^. Another reason could be that input images have been reduced to a low resolution (i.e., 32 × 32 × 32) to accommodate the limitations of the GPU memory possibly leading to a low receptive field from the prediction and robbing the network of sufficient global information.

Semi-automatic tools have been widely used to analyze CCTA images in diagnosing coronary diseases, but several minutes, sometimes more, are necessary for detecting vascular structures because clinical radiologists/technologists must adjust the extracted structure manually. After the manual modification of coronary structures by the semi-automatic tool, clinical radiologists could make a diagnosis from the findings of arterial plaques. Here, an efficient automatic approach was introduced to track individual coronary artery trees. The resulting subjective rating shows that its accuracy coincides with the clinical tool in the main trunk of coronary arteries; it is significantly better in some small branches. The automatic segmentation results based on our approach do not miss small branches such as the conus artery, marginal artery, obtuse marginal two artery, and septal artery while the clinical tool always detects those small branches in the wrong way. Moreover, the automatic segmentation approach proposed here allows rapid extraction of a coronary artery (within 20 s using a 2.7 GHz 4-core CPU with 2 × 8-GB 2133 MHz RAM and one 500-GB SSD). In contrast to the clinical tool, this fully automatic approach not only gives high performance but also great speed in detecting coronary lumen arteries making it a convenience in the clinical diagnosis of coronary diseases.

Though these results are promising, the required computing power and training time are great given that a 3D network is employed. Training requires almost two hours per epoch converging after 40 epochs using a NVIDIA RTX 2080 Ti GPU. Therefore, the tenfold cross validation method was not implemented in this research though it can achieve a slightly better performance. Future research should be focused on reducing computational expense without significantly decreasing performance. Because we focused primarily on addressing the foreground/background imbalance, it would be interesting to apply long short-term memory with a CNN architecture to better understanding the sequential information in CCTA data. Since our method development for automatic coronary artery segmentation is the first step for coronary artery diagnosis, future work should include some processes of centerline detection. MPR will be combined with automatic segmentation for the whole picture of automatic segmentation of calcification and stenosis for clinical diagnosis.

In summary, we developed automatic segmentation of coronary lumen arteries using 3D Dense-U-Net. This is the first step to use AI in diagnosing coronary artery disease. Future work will combine centerline detection and MPR with automatic segmentation of coronary arteries to create completely automatic segmentation of arterial calcifications and stenoses to assist effective clinical diagnoses.
